# Analytic Performance Prediction of Track-to-Track Association with Biased Data in Multi-Sensor Multi-Target Tracking Scenarios

**DOI:** 10.3390/s130912244

**Published:** 2013-09-12

**Authors:** Wei Tian, Yue Wang, Xiuming Shan, Jian Yang

**Affiliations:** 1 Department of Electronic Engineering, Tsinghua University, Beijing 100084, China; E-Mails: wangyue@tsinghua.edu.cn (Y.W.); shanxm@tsinghua.edu.cn (x.S.); yangjian ee@tsinghua.edu.cn (J.Y.); 2 Unit 91715, Navy, People's Liberation Army, Guangzhou 510450, China

**Keywords:** track-to-track association (TTTA), sensor biases, analytic performance prediction, global nearest neighbor (GNN)

## Abstract

An analytic method for predicting the performance of track-to-track association (TTTA) with biased data in multi-sensor multi-target tracking scenarios is proposed in this paper. The proposed method extends the existing results of the bias-free situation by accounting for the impact of sensor biases. Since little insight of the intrinsic relationship between scenario parameters and the performance of TTTA can be obtained by numerical simulations, the proposed analytic approach is a potential substitute for the costly Monte Carlo simulation method. Analytic expressions are developed for the global nearest neighbor (GNN) association algorithm in terms of correct association probability. The translational biases of sensors are incorporated in the expressions, which provide good insight into how the TTTA performance is affected by sensor biases, as well as other scenario parameters, including the target spatial density, the extraneous track density and the average association uncertainty error. To show the validity of the analytic predictions, we compare them with the simulation results, and the analytic predictions agree reasonably well with the simulations in a large range of normally anticipated scenario parameters.

## 1. Introduction

In distributed multi-sensor surveillance systems, the process of associating sets of local estimates from multiple sensors is a fundamental problem, named track-to-track association (TTTA) [1,2]. It is valuable for predicting the performance of TTTA, not only in multi-sensor tracking system design, but also in subsequent situation assessment and on-line resource allocation, such as sensor tasking. Generally, performance evaluation mainly depends on Monte Carlo simulations, which are expensive and time-consuming. Furthermore, little insight can be obtained for the intrinsic relationship between the relevant scenario parameters and the performance based on numerical simulations. Therefore, analytic prediction of data association performance, which does not resort to detailed and costly simulations, is of significant practical and theoretical importance.

With respect to the analytic performance prediction of data association, Sea and Singer did pioneering work on the performance analysis of the nearest neighbor (NN) algorithm [3,4]. Saha provided a methodology for predicting the performance of a logic based ESM/radar TTTA algorithm [5]. Li *et al.* introduced the hybrid conditional averaging technique to predict the performance of the NN tracker [6]. Mei *et al.* gave the formula to calculate the theoretical probability of false association for TTTA using multiscan data [7]. Mori *et al.* derived an analytic expression, named the exponential law, to predict the performance of the global nearest neighbor (GNN) algorithm in terms of the probability of correct association [8,9]. On the basis of Mori's work [8], Ruan *et al.* developed an analytic performance prediction method for the feature-aided GNN algorithm [10]. Some oversimplified assumptions were employed in the derivations, including no false alarm, perfect detection, identical diagonal elements and all other identical elements in the feature confusion matrix, *etc.* Areta *et al.* presented procedures to calculate the probability that the measurement or the track originating from an extraneous target misassociated with a target of interest for the cases of NN and GNN association [11,12]. Mori et al. quantified the utility of additional feature/attribute information for TTTA in [13]. Although these works exhibit various degrees of success, they all do not take the impact of sensor biases on TTTA into consideration.

In performing multi-target tracking with a sensor network, the systematic errors, *i.e.*, sensor biases, can hinder the fusion of data from multiple sensors. Specifically, TTTA is always problematic with biased data, while sensor bias registration usually requires correctly associated data. That is, association and registration actually affect each other [14,15]. To fix this problem, many approaches were proposed to formulate association and registration as a joint optimization problem. However, these joint problems are very difficult to solve, due to the combinatorial nature of the problem and the non-convexity of the objective function [14–19]. Suboptimal solutions are obtained by performing association and registration in an alternately iterative manner. To a certain degree, the prediction of TTTA performance with biased data is also helpful for designing these joint association and registration algorithms.

A number of TTTA algorithms are built upon the GNN criterion [1,2,16,20–23]. Most of them can be decomposed into two steps. The first step is to construct a matrix of association costs (or negative log-likelihood) between two sets of tracks. The second step usually consists of running a linear assignment algorithm on this cost matrix to determine the maximum likelihood association by globally minimizing the cost. The construction of the cost matrix was well discussed in [22–24]. For the second step, many optimal linear assignment algorithms, such as Munkres [25], Jonker-Volgenant [26] and Jonker-Volgenant-Canstenon [27], can be adopted to identify the optimal associations. In this paper, we pay particular attention to predicting the performance of the GNN association algorithm with biased data analytically It should be noted that Castella [28] did consider the influence of biases on the performance of the multiple site track correlation technique proposed by Singer and Kanyuck [29], but the TTTA method of Singer and Kanyuck applies a gating technique and makes the association decision based on the NN rule, rather than the GNN criterion. The goal of our study is to extend the results in [8,9], which established an effective approach for predicting the performance of the optimal assignment algorithms, in an attempt to incorporate the impact of sensor biases.

The rest of the paper is structured as follows. Section 2 describes the single-scan TTTA problem. Section 3 presents the existing analytic prediction results of the bias-free situation. Section 4 provides the analytic prediction results for the GNN association algorithm with biased data. These results are verified via simulations in Section 5. Section 6 concludes the paper.

## 2. Problem Statement

In this paper, we consider a two-sensor single-scan TTTA problem. Although TTTA using multi-scan data has been theoretically proven to be better than that using single-scan data in [7], the former has higher computational complexity and may, in practice, exhibit lower power. That is because using multiscan data increases the degree of freedom of the test statistics, which has a negative impact on the power of the test [30,31]. Therefore, we focus on the single-scan TTTA problem, which is a data association problem with two random point sets in an *m*-dimensional Euclidean space, 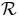
*^m^*.

At a given time (without the time argument, for simplicity), the track set available at sensor A is denoted by ${\left({\text{y}}_{i},{\text{P}}_{A}^{i}\right)}_{i=1}^{N_{A}}$, where **y***_i_* ∈ 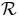
*^m^* and ${\text{P}}_{A}^{i}\in R^{m\times m}$ are the state estimate and error covariance matrix of the *i*-th target at sensor A, respectively; *N_a_* represents the number of tracks available at sensor A. Similarly to sensor A, the track set made by sensor B is ${\left({\text{z}}_{j},{\text{P}}_{B}^{j}\right)}_{j=1}^{N_{B}}$ where **z***_j_* ∈ 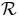
*^m^* and ${\text{P}}_{B}^{j}\in {\text{R}}^{m\times m}$ are the state estimate and error covariance matrix of the *j*-th target at sensor B, respectively; *N_b_* represents the number of tracks available at sensor B. Let y_0_ and z_0_ represent the dummy tracks of sensor A and B, respectively. When a track in a list is not associated with any track from the other list, it will be assigned to the dummy track. Following [8,32], we can define the association between the two track sets as a one-to-one mapping, *a*. Its domain is *Dom*(*a*) ⊆ ℐ = {1,…, *N_A_*}, and its range is *Rng*(*a*) ⊆ 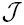 = {1, …, *N_B_*}. For *i* ∈ *Dom*(*a*), *a*(*i*) is the index of the track from sensor B associated with track *i* from sensor A. Tracks in ℐ \ *Dom*(*a*) are associated with z_0_, while tracks in 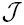 \ *Range*(*a*) are assigned to **y**_0_.

Let a* be the ground-truth association mapping. The track model is given as follows. We assume that sensors have translational biases on their local estimates of targets. The biases are modeled as additive constants. For a common target, *i*, we have:

(1) $${\text{y}}_{i}={\text{x}}_{i}+{\text{b}}_{A}+{\text{u}}_{i},$$

(2) $$z_{a^{*}(i)}=x_{i}+b_{B}+v_{i},$$

where **x***_i_* is the real state of target *i*, **b***_A_*, **b***_B_* represent the biases of sensor A and B, respectively, **u***_i_*, **v***_i_* are the random state estimation errors of target *i* at sensor A and B, respectively, and ${\text{u}}_{i}\sim N\left(0,{\text{P}}_{A}^{i}\right)$, ${\text{v}}_{i}\sim N\left(0,{\text{P}}_{B}^{i}\right)$.

The association can also be defined as an (*N_A_* + 1) ×(*N*_B_ + 1) matrix **h** between ${\left({\text{y}}_{i}\right)}_{i=0}^{N_{A}}$ and ${\left({\text{z}}_{\text{j}}\right)}_{j=0}^{N_{B}}$, with (*i*, *j*) element *h_ij_* for *i* = 1, …, *N_A_* + 1, *j* = 1, …, *N_B_* + 1, such that

(3) $$h_{\text{ij}}=\{\begin{array}{ll} 1, & \text{if}{\text{y}}_{i},{\text{z}}_{j}\;\text{are from the same target}\\ 0, & \text{otherwise} \end{array}.$$

The relationship between *a* and **h** is: if *a*(*i*) = *j*, then *h_ij_* = 1; if *i* ∈ ℐ \ *Dom*(*a*), then *h_i_*_,0_ = 1; if *j* ∈ 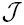 \ *Range*(*a*), then *h*_0,_*_j_* = 1; otherwise *h_i,j_* = 0.

The GNN algorithm determines the best association decision from all possibilities by minimizing the overall cost [1,2]:

(4) $$\underset{\text{h}}{\min}\sum_{i=0}^{N_{A}}\sum_{j=0}^{N_{B}}D_{ij}h_{ij}$$

subject to the constraints:

(5) $$\sum_{i=0}^{N_{A}}h_{\text{ij}}=1,\;j\neq 0$$

(6) $$\sum_{j=0}^{N_{B}}h_{\text{ij}}=1,\;i\neq 0$$

(7) $$h_{\text{ij}}\in \{0,1\}.$$

Constraints (5) and (6) guarantee that each track from a list is assigned to one and at most one track from the other list or to the dummy track. In problem (4), *D_ij_* (*i*, *j* ≠ 0) is the cost between **y***_i_* and **z***_j_*, defined by their normalized distance as:

(8) $$D_{\text{ij}}={\left\|{\text{z}}_{j}-{\text{y}}_{i}\right\|}_{{\text{S}}_{i,j}^{-1}}^{2},$$

where ‖**x**‖_M_ denotes a semi-norm of a vector, **x**, defined by a nonnegative symmetric matrix, **M**, as ${\left\|\text{x}\right\|}_{\text{M}}=\sqrt{{\text{x}}^{T}\text{Mx}}$; **S***_i,j_* is the average association uncertainty covariance matrix and ${\text{S}}_{i,j}={\text{P}}_{A}^{i}+{\text{P}}_{B}^{j}-{\text{P}}_{\text{AB}}^{i,j}-{\text{P}}_{\text{AB}}^{i,j^{T}}$; ${\text{P}}_{AB}^{i,j}$ is the cross-covariance matrix, representing the correlation in the track errors, which results from the common process noise [33]. Because the correlation between tracks is difficult to obtain in practice, one can ignore it by assuming ${\text{P}}_{AB}^{i,j}=0$ [23,24]. The effect of the cross-covariances on TTTA is beyond the scope of this paper, which can be found in [33,34]. We further assume that ${\text{P}}_{A}^{i}={\text{P}}_{A}$ and $P_{B}^{j}=P_{B}$ for all *i* and *j*, such that **S** = **P***_A_* + **P***_B_* is strict positive definite.

In the GNN algorithm, the probability of correct association, *P_C_*, of target *i* is defined as:

(9) $$P_{C}=P\{\hat{a}(i)=a^{*}(i)\},$$

where *â* is the GNN optimal association estimate.

## 3. Previous Results with Bias-Free Data

Mori *et al.* [8,9] derived the analytic expression for the correct association probability, *P_C_*, of the GNN algorithm with bias-free data (*i.e.*, **b***_A_* = 0 in Equation (1), **b***_B_* = 0 in Equation (2)), which obeys an exponential law:

(10) $$P_{C}=\exp(-C_{m}\beta_{T}{\bar{\sigma }}^{m}),$$

under the assumption of no false alarm and perfect detection. Considering the effects of extraneous objects, the expression of *P_C_* was changed into:

(11) $$P_{C}=\exp[-(C_{m}\beta_{T}+D_{m}\beta_{F}){\bar{\sigma }}^{m}].$$

The relevant parameters and assumptions used in Equations (10) and (11) (also effective in the subsequent analysis) are given as follows.

- *C_m_* is a constant defined by:
  (12) $$C_{m}=B_{m}{\tilde{C}}_{m},$$
  where:
  (13) $${\tilde{C}}_{m}=2^{m-1}\pi^{-1/2}\Gamma (\frac{m+1}{2}),$$
  and *B_m_* is the volume of the unit *m*-dimensional ball:
  (14) $$B_{m}=\frac{\pi^{\frac{m}{2}}}{\Gamma \left(\frac{m}{2}+1\right)}.$$
- *β_T_* denotes the spatial density of targets defined as the expected number of targets in a unit volume of the measurement space, 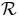
*^m^*:
  (15) $$\beta_{T}=\frac{\lambda_{T}}{B_{m}r^{m}}.$$
  Targets are assumed to be independently identically distributed with a common distribution, which is uniform on a *m*-dimensional ball, with a large enough radius, *r*. The target number, *N_T_*, is a Poisson random variable with a mean, *λ_T_*, that is:
  (16) $$P(N_{T})=e^{-\lambda_{T}}\frac{\lambda_{T}^{N_{T}}}{N_{T}!}.$$
- *D_m_* is a constant given by:
  (17) $$D_{m}=B_{m}{\tilde{D}}_{m},$$
  where:
  (18) $${\tilde{D}}_{m}=2^{m/2}\frac{\Gamma (m)}{\Gamma (m/2)}.$$
- *β_F_* denotes the spatial density of extraneous objects:
  (19) $$\beta_{F}=\frac{\lambda_{F}}{B_{m}r^{m}}.$$
  The states of the extraneous objects share the same distribution with targets. The number of false objects, *N_F_*, is another Poisson random variable with mean *λ_F_*, that is:
  (20) $$P(N_{F})=e^{-\lambda_{F}}\frac{\lambda_{F}^{N_{F}}}{N_{F}!}.$$
- *σ̄* is the average innovation standard deviation.

Actually, the results of Equations (10) and (11) are specified for measurement-to-track association in single-sensor multi-target tracking problems. The novelty of this paper is to extend these results to the TTTA problem with biased data in multi-sensor multi-target tracking applications.

## 4. Analytic Performance Prediction of the GNN Algorithm with Biased Data

In this section, we consider the impact of sensor biases and propose the analytic prediction results for the GNN algorithm.

### 4.1. Association without Extraneous Tracks

First, we start with the two-target case, target *i* and *j* (*i* ≠ *j*). An optimal association, *â*, is correct, i.e., *â*(*i*) = a*(*i*) and *a*(*j*) = a*(*j*), if and only if the cost of the correct association is less than that of the incorrect association, that is:

(21) $$D_{i,a^{*}(i)}+D_{j,a^{*}(j)}\leq D_{i,a^{*}(j)}+D_{j,a^{*}(i)},$$

or, equivalently, if and only if:

(22) $$\Delta J_{\text{ij}}\dot{=}\|{\text{z}}_{a^{*}(i)}-{\text{y}}_{i}{\|}_{{\text{S}}_{i,a^{*}(i)}^{-1}}^{2}+\|{\text{z}}_{a^{*}(j)}-{\text{y}}_{j}{\|}_{{\text{S}}_{j,a^{*}(j)}^{-1}}^{2}-\|{\text{z}}_{a^{*}(j)}-{\text{y}}_{i}{\|}_{{\text{S}}_{i,a^{*}(j)}^{-1}}^{2}-\|{\text{z}}_{a^{*}(i)}-{\text{y}}_{j}{\|}_{{\text{S}}_{j,a^{*}(i)}^{-1}}^{2}$$

(23) $$=-2({\text{z}}_{a^{*}(i)}-{\text{z}}_{a^{*}(j)}){\text{S}}^{-1}({\text{y}}_{i}-{\text{y}}_{j})\leq 0.$$

Considering track model (1) and (2), we have:

(24) $${\text{z}}_{a^{*}(i)}-{\text{z}}_{a^{*}(j)}=({\text{x}}_{i}+{\text{b}}_{B}+{\text{v}}_{i})-({\text{x}}_{j}+{\text{b}}_{B}+{\text{v}}_{j})=({\text{x}}_{i}-{\text{x}}_{j})+({\text{v}}_{i}-{\text{v}}_{j}),$$

(25) $${\text{y}}_{i}-{\text{y}}_{j}=({\text{x}}_{i}+{\text{b}}_{A}+{\text{u}}_{i})-({\text{x}}_{j}+{\text{b}}_{A}+{\text{u}}_{j})=({\text{x}}_{i}-{\text{x}}_{j})+({\text{u}}_{i}-{\text{u}}_{j}).$$

Then, Equation (23) is equivalent to:

(26) $${\left(\Delta {\text{u}}_{\text{ij}}-\Delta {\text{v}}_{\text{ij}}\right)}^{T}{\text{S}}^{-1}\Delta {\text{y}}_{\text{ij}}\leq {\left\|\Delta {\text{y}}_{\text{ij}}\right\|}_{{\text{S}}^{-1}}^{2},$$

where:

(27) $$\Delta u_{\text{ij}}=u_{i}-u_{j},$$

(28) $$\Delta {\text{v}}_{\text{ij}}={\text{v}}_{i}-{\text{v}}_{j},$$

(29) $$\Delta {\text{y}}_{\text{ij}}={\text{y}}_{i}-{\text{y}}_{j}.$$

Thus, based on the detailed derivations in Appendix A, the probability of correct association with two targets is:

(30) $$P_{C}=P\{\hat{a}(i)=a^{*}(i)\}=P\{\Delta J_{\text{ij}}\leq 0\}\approx 1-{\tilde{C}}_{m}{\left(\frac{\bar{\sigma }}{r}\right)}^{m},$$

where *σ̄* is the average association standard deviation, and:

(31) $$\bar{\sigma }={\left[\text{det}(S)\right]}^{\frac{1}{2m}}.$$

It is very difficult to extend Equation (30) to the multi-target case, because the incorrect associations may have more complicated errors besides the two-object transposition, such as the multi-object transposition. To overcome this difficulty, let the event, *E_C_*{*â*(*i*) = a*(*i*)} (target *i* is correctly associated), be approximated by the event that there is no two-object transposition of Δ*J_ij_* > 0 involving target *i*. With Equation (16) and a further assumption that the event {Δ *J_ij_* > 0} is independent for each *j*, *i.e.*, each “potential” transposition is independent, we have:

(32) $$P_{C}=\sum_{N_{T}=0}^{\infty }P\{\hat{a}(i)=a^{*}(i)|N_{T}\}|P(N_{T})$$

(33) $$=\sum_{N_{T}=0}^{\infty }\prod_{j=1,j\neq i}^{N_{T}}P\{\Delta J_{\text{ij}}\leq 0\}P(N_{T})$$

(34) $$=\sum_{N_{T}=0}^{\infty }{\left[1-{\tilde{C}}_{m}{\left(\frac{\bar{\sigma }}{r}\right)}^{m}\right]}^{N_{T}-1}P(N_{T})$$

(35) $$=e^{-\lambda_{T}}\frac{\sum_{N_{T}=0}^{\infty }{\left[1-{\tilde{C}}_{m}{\left(\frac{\bar{\sigma }}{r}\right)}^{m}\right]}^{N_{T}}\frac{\lambda_{T}^{N_{T}}}{N_{T}!}}{1-{\tilde{C}}_{m}{\left(\frac{\bar{\sigma }}{r}\right)}^{m}}$$

(36) $$={\tilde{C}}_{m}^{\prime }\text{exp}(-{\tilde{C}}_{m}{\tilde{\beta }}_{T})$$

(37) $$={\tilde{C}}_{m}^{\prime }\text{exp}(-C_{m}\beta_{T}{\bar{\sigma }}^{m}),$$

where:

(38) $${\tilde{C}}_{m}^{\prime }=\frac{1}{1-{\tilde{C}}_{m}{\left(\frac{\bar{\sigma }}{r}\right)}^{m}};$$

and:

(39) $${\tilde{\beta }}_{T}=B_{m}\beta_{T}{\bar{\sigma }}^{m}$$

is the normalized target density, which is defined as the expected number of targets in the one-sigma ellipsoidal volume derived from the average association uncertainty covariance matrix, **S**. The formula:

(40) $$e^{x}=\sum_{i=0}^{+\infty }\frac{x^{i}}{i!}$$

is used in the derivation of Equation (36). The idea of independent transpositions was first introduced in [35], which gave some justification of this assumption. Generally, all possible association mappings are considered to be independent with each other and totally random. In this sense, the independent transposition assumption is reasonable.

Generally, *r* ≫ *σ̄*. Hence:

(41) $${\tilde{C}}_{m}^{\prime }\approx 1,$$

which was used in the derivation of Equation (10) in [8]. Consequently, Equation (37) becomes

(42) $$P_{C}\approx \text{exp}(-C_{m}\beta_{T}{\bar{\sigma }}^{m}).$$

To maintain consistency with [8–10], Equation (42) is used in the following discussions. Since the bias terms are canceled out in Equations (24) and (25), Equation (42) is the same as (10), which does not account for sensor biases. Then, we can see that without extraneous and missed tracks, the translational biases do not affect the performance of the GNN association algorithm.

### 4.2. Extraneous Tracks Only Existing in One Track Set

In Section 4.1, we assumed that the cardinalities of the two given random point sets are identical. In reality, false tracks may disturb the situation. In this section, we discuss the effects of false tracks on the TTTA performance with biased data, in which there are false tracks only in the track set of sensor B. The event, *E_C_*{*â*(*i*) = a*(*i*)}, can be written as

(43) $$E_{C}\{\hat{a}(i)=a^{*}(i)\}\approx E_{C_{T}}\cap E_{C_{F}},$$

where *E_CT_* is the event that there is no transposition between target *i* and any other target and *E_C_f__* is the event that target track **y***_i_* is not associated with any false tracks. Since the targets and false alarms are independent from each other, we have:

(44) $$P_{C}\dot{=}P(E_{C}\{\hat{a}(i)=a^{*}(i)\})\approx P(E_{C_{T}})P(E_{C_{F}}).$$

We can use the result of Equation (42) for *P*(*E_C_T__*). The focus now is to analyze *P*(*E_C_F__*) in the situation where sensors have translational biases.

Given an extraneous track, **z***_e_*, and the target track, **z**_a^*^(i)_, from sensor **B**, let *P_ie_* be the probability that **z***_e_* is not associated with **y***_i_*, that is:

(45) $$P_{\text{ie}}=P\{\|{\text{z}}_{a^{*}(i)}-{\text{y}}_{i}{\|}_{{\text{S}}^{-1}}^{2}\leq \|{\text{z}}_{e}-{\text{y}}_{i}{\|}_{{\text{S}}^{-1}}^{2}\}.$$

Without loss of generality, let **y***_i_* = 0. Then, Equation (45) is equivalent with:

(46) $$P_{\text{ie}}=P\{\|{\text{z}}_{a^{*}(i)}{\|}_{{\text{S}}^{-1}}^{2}\leq \|{\text{z}}_{e}{\|}_{{\text{S}}^{-1}}^{2}\}.$$

According to the track model (1), we can get the real state of target *i*, for the form only, as:

(47) $${\text{x}}_{i}=-{\text{b}}_{A}-{\text{u}}_{i}.$$

Consequently, together with Equations (2) and (47), we have:

(48) $${\text{z}}_{a^{*}(i)}=-{\text{b}}_{A}-{\text{u}}_{i}+{\text{b}}_{B}+{\text{v}}_{i}=\Delta \text{b}+{\text{v}}_{i}-{\text{u}}_{i},$$

where:

(49) $$\Delta b=b_{B}-b_{A},$$

is the relative translational bias vector between the two sensors. Then, based on Equations (1), (2) and (48), we have **z**_a_*_(_*_i_*_)_ ˜ 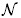
(Δ**b**, **S**). Thus, ${\left\|{\text{z}}_{a^{*}(i)}\right\|}_{{\text{S}}^{-1}}^{2}$ is distributed according to the noncentral chi-squared distribution with the degree of freedom, *m*, and the noncentrality parameter, *δ*, i.e., ${\left\|{\text{z}}_{a^{*}(i)}\right\|}_{{\text{S}}^{-1}}^{2}\sim \chi_{m}^{2}(\delta )$ [36]. The noncentrality parameter, *δ*, is:

(50) $$\delta =\Delta {\text{b}}^{T}{\text{S}}^{-1}\Delta \text{b}.$$

Assuming that the extraneous track, **z***_e_*, is uniformly distributed on the *m*-dimensional ball with radius *r*, the distribution of ${\left\|z_{e}\right\|}_{S^{-1}}^{2}$ can be expressed as:

(51) $$P\{{\left\|{\text{z}}_{e}\right\|}_{{\text{S}}^{-1}}^{2}<y\}={\left(\frac{\sqrt{y}}{\frac{r}{\bar{\sigma }}}\right)}^{m}.$$

Let $Y={\left\|z_{e}\right\|}_{S^{-1}}^{2}$; then, its probability density function is

(52) $$f_{Y}(y)=\frac{\partial P(Y<y)}{\partial y}=\frac{m/2}{{\left(\frac{r}{\bar{\sigma }}\right)}^{m}}y^{m/2-1}.$$

Let $X={\left\|{\text{z}}_{a^{*}(i)}\right\|}_{{\text{S}}^{-1}}^{2}$ and the probability density function of *X* be denoted by *f_X_* (*x*; *m*, *δ*). Considering that the extraneous track, **z***_e_*, and the target track, **z**_a^*^(i)_, are independent, the joint probability density function of random variables, *X* and *Y*, can be calculated as:

$$f_{X,Y}(x,y)=f_{X}(x)f_{Y}(y)=f_{X}(x;m,\delta )f_{Y}(y).$$

Then, we have:

(53) $$P_{\text{ie}}=P\{X\leq Y\}$$

(54) $$=\int \int_{\left\{(x,y):x\leq y\right\}}f_{X,Y}(x,y)\text{dxdy}$$

(55) $$=\int_{-\infty }^{+\infty }\int_{x}^{+\infty }f_{X}(x)f_{Y}(y)\text{dydx}$$

(56) $$=\int_{-\infty }^{+\infty }\int_{x}^{{\left(\frac{r}{\bar{\sigma }}\right)}^{2}}f_{X}(x;m,\delta )\frac{m/2}{\left(\frac{r}{\bar{\sigma }}\right)m}y^{m/2-1}\text{dydx}$$

(57) $$=\frac{m/2}{{\left(\frac{r}{\bar{\sigma }}\right)}^{m}}\int_{-\infty }^{+\infty }f_{X}(x;m,\delta )\int_{x}^{{\left(\frac{r}{\bar{\sigma }}\right)}^{2}}y^{m/2-1}\text{dydx}$$

(58) $$=\frac{m/2}{{\left(\frac{r}{\bar{\sigma }}\right)}^{m}}\int_{-\infty }^{+\infty }f_{X}(x;m,\delta )\frac{\left({\left(\frac{r}{\bar{\sigma }}\right)}^{m}-x^{m/2}\right)}{m/2}\text{dx}$$

(59) $$=\frac{1}{{\left(\frac{r}{\bar{\sigma }}\right)}^{m}}[\int_{-\infty }^{+\infty }f_{X}(x;m,\delta ){\left(\frac{r}{\bar{\sigma }}\right)}^{m}\text{dx}-\int_{-\infty }^{+\infty }x^{m/2}f_{X}(x;m,\delta )\text{dx}]$$

(60) $$=1-\frac{1}{{\left(\frac{r}{\bar{\sigma }}\right)}^{m}}\int_{-\infty }^{+\infty }x^{m/2}f_{X}(x;m,\delta )\text{dx}$$

(61) $$=1-\frac{1}{{\left(\frac{r}{\bar{\sigma }}\right)}^{m}}\varepsilon_{\chi_{m}^{2}(\delta )}\left(x^{m/2}\right)$$

(62) $$=1-{\tilde{D}}_{m}^{\prime }{\left(\frac{\bar{\sigma }}{r}\right)}^{m},$$

where:

(63) $${\tilde{D}}_{m}^{'}=\varepsilon_{\chi_{m}^{2}(\delta )}(x^{m/2})=\int_{-\infty }^{+\infty }x^{m/2}f_{X}(x;m,\delta )dx.$$

If *m* = 2*k* and *k* is a positive integer, Equation (63) becomes

(64) $${\tilde{D}}_{m}^{'}=\varepsilon_{\chi_{m}^{2}(\delta )}(x^{k}),$$

which is the *k*th order moment of the noncentral chi square distribution with degree of freedom *m* and noncentral parameter *δ*. If *m* = 2*k* + 1 and *k* is a nonnegative integer, Equation (63) is the fractional moment of the noncentral chi square distribution, and the calculation method of Equation (63) can be found in [37].

When there are *N_F_* false tracks in the track set of sensor B, with the independence assumption, the probability that no extraneous track is associated with **y***_i_* is

(65) $$P(E_{C_{F}}|N_{F})=P_{\text{ie}}^{N_{F}}.$$

Under the assumption of the false track number in Equation (20), we have:

(66) $$P(E_{C_{F}})=e^{-\lambda_{F}}\sum_{N_{F}=0}^{\infty }\frac{\lambda_{F}^{N_{F}}}{N_{F}!}P_{\text{ie}}^{N_{F}}$$

(67) $$=\text{exp}[-\lambda_{F}(1-P_{\text{ie}})]$$

(68) $$=\text{exp}[-\lambda_{F}{\tilde{D}}_{m}^{'}{\left(\frac{\bar{\sigma }}{r}\right)}^{m}]$$

(69) $$=\text{exp}[-{\tilde{\beta }}_{F}{\tilde{D}}_{m}^{'}]$$

(70) $$=\text{exp}[-B_{m}\beta_{F}{\bar{\sigma }}^{m}{\tilde{D}}_{m}^{'}]$$

(71) $$=\text{exp}[-D_{m}^{'}\beta_{F}{\bar{\sigma }}^{m}],$$

where

(72) $${\tilde{\beta }}_{F}=B_{m}\beta_{F}{\bar{\sigma }}^{m}$$

is the normalized density of false tracks, and

(73) $$D_{m}^{'}=B_{m}{\tilde{D}}_{m}^{'}.$$

By substituting Equations (42) and (71) into Equation (44), we have:

(74) $$P_{C}=\text{exp}(-C_{m}\beta_{T}{\bar{\sigma }}^{m})\text{exp}[-D_{m}^{'}\beta_{F}{\bar{\sigma }}^{m}]$$

(75) $$=\text{exp}\{-[{\tilde{C}}_{m}{\tilde{\beta }}_{T}+{\tilde{D}}_{m}^{'}{\tilde{\beta }}_{F}]\}$$

(76) $$=\text{exp}\{-[C_{m}\beta_{T}+D_{m}^{'}\beta_{F}]{\bar{\sigma }}^{m}\}.$$

When there are no extraneous tracks, *i.e.*, *β*_F_ = 0, Equation (76) is the same with Equation (42). The effect of sensor biases is coupled with false tracks by the multiplying factor, $D_{m}^{'}(\text{or}\;{\tilde{D}}_{m}^{'})$. With *m* = 2, we have *C_m_* = *π*, $D_{m}^{'}=\pi (2+\delta )$; then:

(77) $$P_{C}=\text{exp}\{-\pi [\beta_{T}+\beta_{F}(2+\delta )]{\bar{\sigma }}^{2}\}.$$

In addition, if sensors do not have biases (or have the same translational biases), i.e., Δ**b** = 0, then δ = 0, $D_{m}^{'}=2\pi$, and

(78) $$P_{C}=\text{exp}\{-\pi [\beta_{T}+2\beta_{F}]{\bar{\sigma }}^{2}\},$$

which is consistent with Equation (11) under the two-dimensional case.

### 4.3. Extraneous Tracks Existing in Both Track Sets

When false tracks exist in both track sets, with a simple generalization of Equation (43), we have:

(79) $$E_{C}\{\hat{a}(i)=a^{*}(i)\}\approx E_{C_{T}}\cap E_{C_{FA}}\cap E_{C_{FB}},$$

where *E_C_FA__* is the event that track **y***_i_* is not associated with any false track from sensor B and *EC_FB_* is the event that track **z***_j_* is not associated with any false track from sensor A. With the assumption that these events are independent, Equation (44) then is extended to:

(80) $$P_{C}\approx P(E_{C_{T}})P(E_{C_{\text{FA}}})P(E_{C_{\text{FB}}})$$

(81) $$=\text{exp}\left\{-\left[{\tilde{C}}_{m}{\tilde{\beta }}_{T}+{\tilde{D}}_{m}^{'}\left({\tilde{\beta }}_{\text{FA}}+{\tilde{\beta }}_{\text{FB}}\right)\right]\right\}$$

(82) $$=\text{exp}\left\{-\left[C_{m}\beta_{T}+D_{m}^{'}\left(\beta_{\text{FA}}+\beta_{\text{FB}}\right)\right]{\bar{\sigma }}^{m}\right\},$$

where *β_FA_* and *β_FB_* are the densities of false tracks made by sensor A and B, respectively; ${\tilde{\beta }}_{\text{FA}}$ and ${\tilde{\beta }}_{\text{FB}}$ are the normalized densities of false tracks made by sensor A and B, respectively.

Comparing Equation (82) with (76), it seems that the situation with false tracks in both track sets has the same difficulty with that in only one sensor. However, this is not true. As shown in Section 5.3, the relative quantities of false tracks between sensors have a large impact on the *P_C_*; so, the simple sum of *β_FA_*+ *β_FB_* in Equation (82) is not sufficient to reflect the intrinsic impact of false tracks on the correct association probability. Other risks in analytic performance prediction using Equation (82) will be examined through simulations in Section 5.3. Here, we just give the main conclusion that in a large range of normally anticipated operating conditions, Equation (82) can be used as an upper bound for the performance of the GNN association algorithm in terms of the correct association probability.

## 5. Simulation

To show the validity of our theoretical analysis, we test the above analytic results by comparing them to the simulation results. We consider the single-scan TTTA problem and make use of the Munkres algorithm to solve the GNN assignment problem [25]. The scenario consists of two sensors tracking multiple targets. The targets are distributed uniformly in a two-dimensional ball space (*m* = 2) with target extent radius *r*. The number of targets is a Poisson variable with mean λ*_T_* = 20, and the target spatial density is varied by changing *r*. The random error covariance matrices of the target state estimates are presumed to satisfy **P***_A_* = **P***_B_*. The correlation among tracks from different sensors due to the common process noise is neglected in the following simulations, and the detailed analysis and simulations for the correlated tracks can be found in [33,34]. Sensors may report extraneous tracks, which are assumed to have the same spatial distribution with the target state. The track detection probability of each sensor is presumed to be one. Since the translational biases are in Cartesian coordinates, only the difference of them is observable. Therefore, the relative biases between sensors, instead of the absolute biases of each sensor, are considered in simulation. Let Δ*b_x_*, Δ*b_y_* represent the relative translational biases in the x,y coordinate, respectively. All of the simulation results are based upon 500 Monte Carlo runs.

### 5.1. No Extraneous Tracks

**Figure 1.**
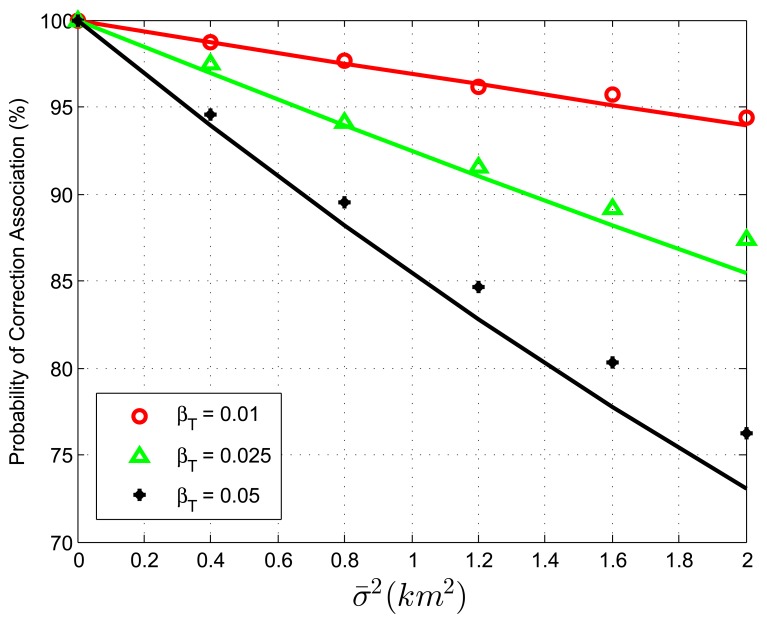
Correct association probability as a function of the average association uncertainty variance—analytic prediction results against simulation results. Target spatial density *β_T_* is 0.01, 0.025, 0.05 *reports*/*km*^2^. Sensor biases are set as Δ*b_x_* = Δ*b_y_* = 1 *km*.

When we study the validity of Equation (42) with biased data and no extraneous tracks, two simulation experiments are designed. The target spatial density, *β_T_*, is set to be 0.01, 0.025, 0.05 *reports*/*km*^2^, with the target extent radius *r* being 25.23, 15.95, 11.28 *km*, respectively. In the first experiment, the average association uncertainty covariance matrix is fixed to be **S** = *diag*[1, 1] *km*^2^, while Δ*b_x_*, Δ*b_y_* vary from −3 *km* to 3 *km* in increments of 1 *km*. Tables 1, 2 and 3 show that the correct association probability, *P_C_*, obtained by simulations remains approximately unchanged and matches the analytic prediction well, even if the sensor biases vary in a big range. That is because, when no extraneous tracks exist, the *P_C_* of the GNN association algorithm is not associated with translational biases in Equation (42). From the comparison between Tables 1, 2 and 3, the theoretical *P_C_* value given by Equation (42) is less accurate as the track density increases. An explanation of this phenomenon is that, although the assumption of a large enough *r* is necessary in the derivation of Equation (42), the target extent radius, *r*, is not large enough under a high target density. Another possible reason might originate from the more complicated association errors, i.e., multi-object transpositions, which we did not model. However, the statistics concerning such “high-order” transpositions are not clear at the moment. In the second experiment, sensor biases are fixed to be Δ*b_x_* = Δ*b_y_* = 1 *km*, while the average association variance, *σ̄*^2^, varies from 0 *km*^2^ to 2 *km*^2^ in increments of 0.4 *km*^2^. Figure 1 plots the correct association probability, *P_C_*, as a function of *σ̄*^2^, and each point is compared with the prediction (solid line) by Equation (42). The analytic prediction results match the simulation results very well, with a few exceptions: when the targets are dense and the average association variance is large. In view of the approximations we have employed, including some quite “bold” ones, such as the independent transposition assumption, this consistency between the simulation results and the theoretical prediction is rather remarkable.

### 5.2. Extraneous Tracks Only in One Track Set

**Figure 2.**
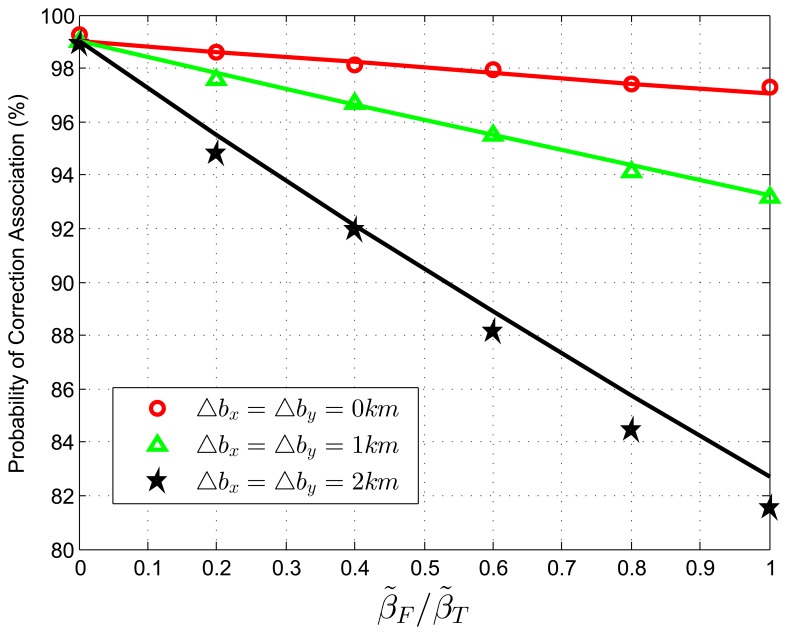
Correct association probability as a function of *β˜_F_*/*β˜_T_*—analytic prediction results against simulation results. The normalized target spatial density, *β˜_T_*, is fixed to be 0.05 *reports*. Sensor biases are Δ*b_x_* = Δ*b_y_* = 0,1,2 *km*.

When we test Equation (76) with extraneous tracks in the track set of sensor B, the normalized target density, *β˜_T_*, is fixed to be 0.05 *reports*, with the average association uncertainty covariance matrix, **S** = *diag*[0.5, 0.5] *km*^2^, and the target extent radius, *r* = 14.14 *km*. The relative translational biases are set as Δ*b_x_* = Δ*b_y_* = 0, 1, 2 *km*. The ratio, *β˜_F_*/*β˜_T_*, is changed from zero to one in increments of 0.2. In each Monte Carlo run, false tracks are added according to given densities. Figure 2 shows the correct association probability as a function of the ratio, *β˜_F_*/*β˜_F_*, which determines the relative density of extraneous tracks to target tracks. The analytic prediction results (solid lines) match the simulation results (points) very well, and they perform better matching when small sensor biases occur.

### 5.3. Extraneous Tracks in Both Track Sets

**Figure 3.**
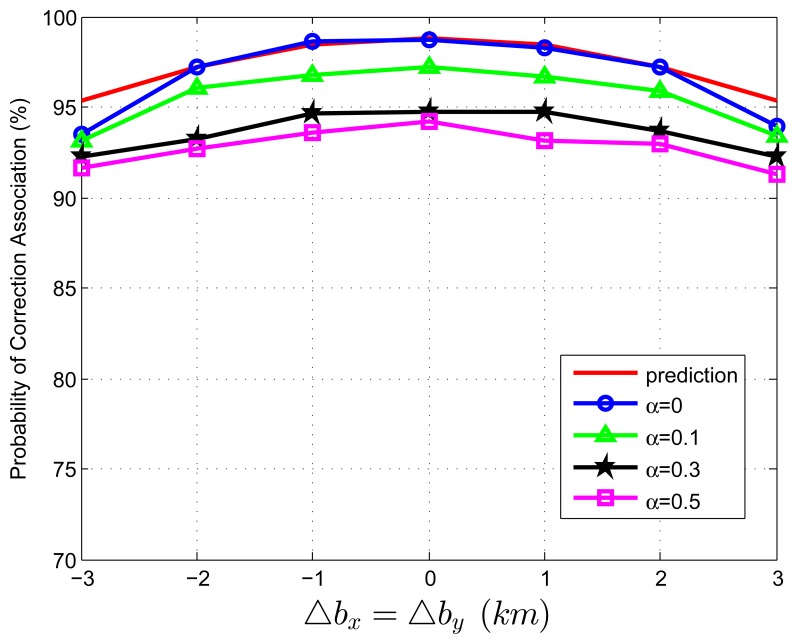
Correct association probability as a function of sensor biases when both track sets contain extraneous tracks—analytic prediction results *versus* simulation results. The normalized target spatial density, *β˜_T_*, is fixed to be 0.01 *reports*. The total normalized false track density is set as *β˜_F_* = 0.1*β˜_T_*.

**Figure 4.**
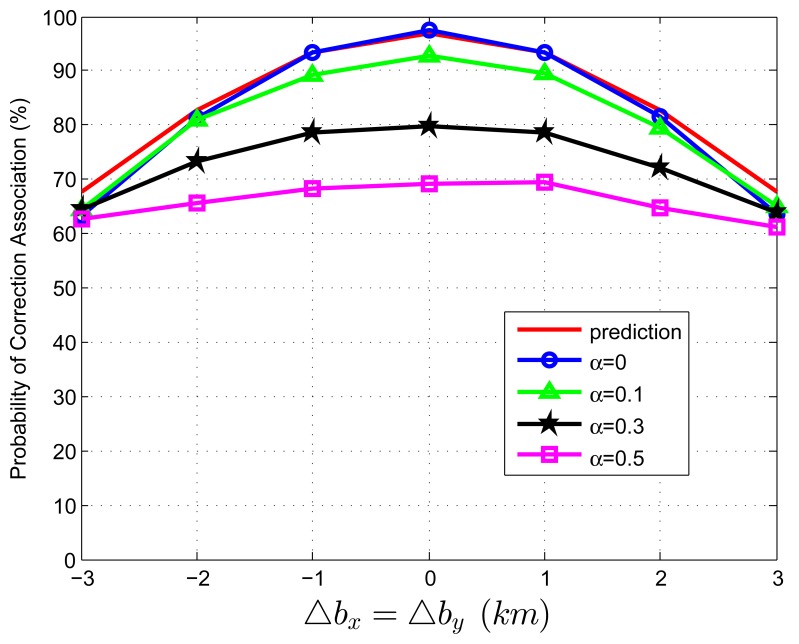
Correct association probability as a function of sensor biases when both track sets contain extraneous tracks—theoretical prediction *versus* simulation results. The normalized target spatial density, *β˜_T_*, is fixed to be 0.01 *reports*. The total normalized false track density is set as *β˜_F_* = *β˜_T_*.

**Figure 5.**
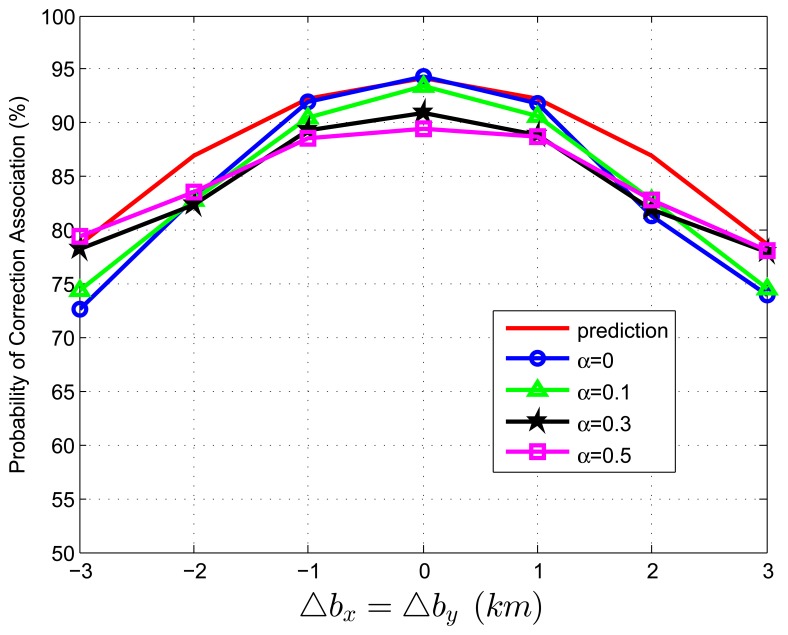
Correct association probability as a function of sensor biases when both track sets contain extraneous tracks—analytic prediction results *versus* simulation results. The normalized target spatial density, *β˜_T_*, is fixed to be 0.05 *reports*. The total normalized false track density is set as *β˜_F_* = 0.1*β˜_T_*.

**Figure 6.**
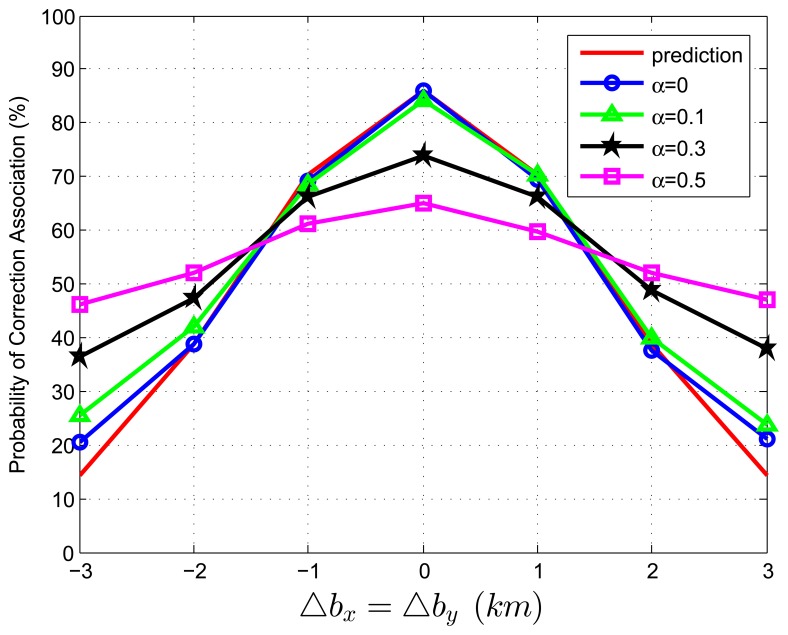
Correct association probability as a function of sensor biases when both track sets contain extraneous tracks—analytic prediction results *versus* simulation results. The normalized target spatial density, *β˜_T_*, is fixed to be 0.05 *reports*. The total normalized false track density is set as *β˜_F_* = *β˜_T_*.

When we test Equation (82) with extraneous tracks in both track sets, the average association covariance matrix is set to be **S** = *diag*[0.5, 0.5] *km*^2^, and Δ*b_x_* is assumed to be equal with Δ*b_y_* varying from -3 *km* to 3 *km* in increments of 1 *km*. Let *β˜_F_* be the total normalized density of extraneous tracks, and *β˜_F_* = *β˜_FA_* + *β˜_FB_*. Let *α* be the ratio of false tracks from sensor A, and *α* = *β˜_FA_*/*β˜_F_*. Given the total density of false tracks, the parameter, *α*, reflects the allocation (or the relative quantity) of the extraneous tracks between sensor A and B. Four cases of *α* (*α* = 0, 0.1, 0.3, 0.5) are considered in the simulations. To better understand the prediction performance of Equation (82), two groups of simulation experiments are performed. In the first group, the normalized target spatial density, *β˜_T_*, is fixed to be 0.01 *reports* (accordingly, *r* is set as 31.62 *km*). Figures 3 and 4 plot the correct association probability as a function of sensor biases, with *β˜_F_* = 0.1*β˜_T_* and *β˜_F_* = *β˜_T_*, respectively. In the second group, *β˜_T_* is fixed to be 0.05 *reports* (accordingly, *r* is 14.14 *km*). Figures 5 and 6 plot the correct association probability as a function of sensor biases, with *β˜_F_* = 0.1*β˜_T_* and *β˜_F_* = *β˜_T_*, respectively.

Since a series of assumptions are made in the derivation of Equation (82), the prediction accuracy degrades in the scenario where false tracks exist in both sensors. When the number of extraneous objects is far less than the number of targets (*β˜_F_* = 0.1*β˜_T_*), the prediction results are an upper bound for the simulation results, and the prediction deviation is below 7%, as shown in Figures 3 and 5. While there are a lot of false tracks (*β˜_F_* = *β˜_T_*) in Figures 4 and 6, Figure 4 shows that, in the sparse-target scenario (*β˜_T_* = 0.01 *reports*), even with lots of false tracks, Equation (82) can also be used as an upper bound for the simulation results. Comparing Figure 6 with Figures 3, 4 and 5, we can see that this prediction upper bound becomes invalid only in the scenario with a high target density (*β˜_T_* = 0.05 *reports*), a high false track density (*β˜_F_* = *β˜_T_*) and large sensor biases (| Δ*b_x_*| > 1.3*km*). Meanwhile, the prediction departure becomes larger as *α* increases. The fundamental reason for the prediction departure is not clear at this point. Two possible explanations are given as follows. First, in this complicated scenario, especially with a large *α* (≤ 0.5), false tracks of different sensors may associate with each other. This leads to a “reduction” of the false track density. In this sense, *β_FA_* + *β_FB_* in Equation (82) overestimates the density of false tracks. In addition, this false track “reduction” effect can be amplified under large sensor biases, since the multiplying factor, $D_{m}^{\prime }$, in Equation (82) is a quadratic function of sensor biases. Second, when both track sets have false tracks, the multi-object transpositions we did not model are apt to occur. Yet, for all that, under large sensor biases and a large *α* (in Figure 6), the extreme pessimistic tendency of the prediction does not matter very much, because it appears only when *P_C_* is quite small (below 50%).

To sum up, in a large range of normally anticipated operating conditions, Equation (82) can be used as an upper bound for the simulation results. Further investigations are needed to provide a more accurate prediction of TTTA in the cases where false tracks exist in both sensors.

## 6. Conclusion

In this paper, we proposed an analytic performance prediction method for the GNN association algorithm with biased data in multi-sensor multi-target tracking applications. The probability of correct association is adopted as the performance criteria. The novelty of the method lies in that it accounts for the sensor biases in the TTTA problem, and analytic approaches are developed to reveal the intrinsic relationship between a set of key scenario parameters and the performance of the optimal TTTA algorithm. To verify the validity of the predictions, we compared them with the results of Monte-Carlo simulations. These shows that the analytic predictions agree reasonably well with the simulation results. In a word, the main results of the paper are as follows:

(1) When both track sets are free from extraneous tracks, the correct association probability of the GNN association algorithm in Equation (42) has nothing to do with the translational biases of sensors. It is only related with target spatial density and the average association errors.
(2) When one track set suffers from extraneous tracks, an exponential law was given in Equation (76), which uncovers the relationship between the correct association probability and the scenario parameters, including the average association errors, sensor biases, the target spatial density, as well as the false track density. The impact of sensor biases is embodied by an amplification coefficient of the false track density.
(3) When both track sets contain extraneous tracks, an analytic upper bound of the correct association probability for the GNN association algorithm was proposed in Equation (82), which is effective in a large range of scenario parameters.

A series of assumptions were made to get concise and explicit mathematical results, including no missed detection, the translational bias model, identical association uncertainty covariance on all tracks, *etc.* The results derived based on these assumptions are not intended to be used directly in practice. The major contribution of the paper is to help designers understand the intrinsic relationship between a set of key scenario parameters and the performance of TTTA and to demonstrate the possibility that analytic performance prediction can be a potential substitute for the costly Monte Carlo simulation method.

To make the proposed analytic method more practical, a lot of work is needed in the following directions:

(1) Further investigations are needed to improve the prediction accuracy of our method in the scenario where false tracks exist in both sensors. The statistics concerning the more complicated association error,s such as multi-object transpositions, can be addressed in the future.
(2) For simplicity, we assumed that the track detection probabilities of both sensors are one. However, missed detections and false tracks do happen in real environments. To complicate matters, when missed detections occur at one sensor, the corresponding tracks that are supposed to associate with these missed tracks in the track set of the other sensor are equivalent to extraneous tracks for the remaining tracks of the former sensor. When sensors suffer from both missed detections and extraneous tracks, accounting for the missed detections in the formula of the correct association probability needs to be investigated in the future.
(3) We only examined the impact of translational biases on the performance of TTTA. Since the impact of translational biases on the tracks is uncoupled with the states of targets, the translational biases can be named state-independent biases. However, in some applications, sensors can have biases on their range and azimuth measurements. The impact of these biases on the position estimates of targets is dependent on the real states of targets. Therefore, we name the range and azimuth biases the state-dependent biases. Under the scenario with state-dependent biases, the bias terms cannot be canceled out in Equations (24) and (25). Thus, the state-dependent biases affect the performance of the GNN association algorithm, even if there are no false tracks and missed detections. How to deal with the more complicated state-dependent biases in analytic performance prediction is left for the future.

## Figures and Tables

**Table 1.** The impact of sensor biases on correct association probability—the prediction by Equation (42) is 96.90%. *β_T_* = 0.01 *reports*/*km*^2^.

P_C_ (%)		Δb_x_						
		−3km	−2km	−1km	0km	1km	2km	3km
Δ*b_y_*	-3 km	96.87	97.16	96.63	97.23	97.48	97.55	97.09
	-2 km	96.85	96.86	97.03	97.23	97.26	97.10	97.06
	-1 km	97.21	96.80	96.37	96.91	96.62	96.95	97.00
	0 km	97.43	97.37	96.79	97.24	97.26	97.43	96.95
	1 km	97.19	97.04	97.67	97.52	97.11	97.16	97.17
	2 km	97.07	97.20	97.17	96.90	97.62	97.67	97.32
	3 km	97.26	97.08	97.28	96.88	97.19	97.17	97.11

**Table 2.** The impact of sensor biases on correct association probability—the prediction by Equation (42) is 92.44%. *β_T_* = 0.025 *reports*/*km*^2^.

P_C_ (%)		Δb_x_						
		−3km	−2km	−1km	0km	1km	2km	3km
Δ*b_y_*	-3 km	92.98	93.14	93.07	93.36	93.47	92.96	92.5
	-2 km	93.54	92.98	93.55	93.03	93.25	93.18	93.27
	-1 km	92.87	94.02	92.83	93.15	93.35	92.98	92.21
	0 km	93.03	92.41	93.30	93.21	93.00	93.02	92.89
	1 km	92.14	93.67	92.87	92.95	93.44	93.44	93.92
	2 km	93.17	92.80	92.40	93.11	93.24	92.76	93.15
	3 km	92.87	92.91	93.58	92.83	93.14	93.17	92.77

**Table 3.** The impact of sensor biases on the correct association probability—the prediction by Equation (42) is 85.46%. *β_T_* = 0.05 *reports*/*km*^2^.

P_C_ (%)		Δb_x_						
		−3km	−2km	−1km	0km	1km	2km	3km
Δ*b_y_*	-3 km	87.81	86.85	87.20	87.79	86.73	87.03	86.62
	-2 km	87.51	86.83	86.48	87.34	86.31	86.29	87.63
	-1 km	86.88	87.78	86.66	86.63	86.61	87.28	86.25
	0 km	87.24	87.07	86.39	88.13	87.64	86.46	86.98
	1 km	87.17	87.01	87.10	86.63	87.02	86.95	88.18
	2 km	86.73	86.68	86.74	87.27	86.42	87.10	86.86
	3 km	86.48	86.49	87.28	86.85	86.57	87.34	86.71

## Appendix A

In this appendix, we give the derivation of Equation (30). Most of the results in this appendix are taken from [8].

(83) $$P_{C}=P\{\hat{a}(i)=a^{*}(i)\}=P\{\Delta J_{\text{ij}}\leq 0\}$$

(84) $$=\varepsilon [P({\left(\Delta {\text{u}}_{\text{ij}}-\Delta {\text{v}}_{\text{ij}}\right)}^{T}{\text{S}}^{-1}\Delta {\text{y}}_{\text{ij}}\leq {\left\|\Delta {\text{y}}_{\text{ij}}\right\|}_{{\text{S}}^{-1}}^{2})|\Delta {\text{y}}_{\text{ij}}]$$

(85) $$=\varepsilon (\text{erf}(\alpha_{\text{ij}}))$$

where $\alpha_{\text{ij}}=\left\|\Delta {\text{y}}_{\text{ij}}\right\|{\text{s}}^{-1}/\sqrt{2}$, ε[·] is the mathematical expectation operator, *erf*(·) is the error function defined as $\text{erf}(c)≐\frac{1}{2\pi }\int_{-\infty }^{c}e^{-t^{2}/2}\text{dt}$ and its complement is denoted by *erfc*(*c*), *i.e.*, *erfc*(*c*) = 1 − *erf*(*c*).

Consider the case where target *i* is located at the origin, while target *j* is at a random position according to the uniform distribution within the *m*-ball with radius *r*. Then, we have:

(86) $$\varepsilon (\text{erf}(\alpha_{\text{ij}})|{\text{y}}_{i}=0)=1-{\left(B_{m}r^{m}\right)}^{-1}\int_{\left\|\Delta {\text{y}}_{\text{ij}}\right\|\leq r}\text{erfc}(\alpha_{\text{ij}})d\Delta {\text{y}}_{\text{ij}}$$

(87) $$=1-{\left(B_{m}r^{m}\right)}^{-1}2^{m/2}{\left[\text{det}(\text{S})\right]}^{1/2}\int_{D(r)}\text{erfc}(\left\|\eta \right\|)d\eta$$

(88) $$\approx 1-B_{m}^{-1}2^{m/2}{\left(\frac{\bar{\sigma }}{r}\right)}^{m}\int_{R^{m}}\text{erfc}(\left\|\eta \right\|)d\eta ,$$

where $D(r)≐\{\eta \in R^{m}|\sqrt{2\eta^{T}\text{S}\eta }\leq r\}$; the approximation was made by replacing (*r*) with *^m^*, assuming that *r* is large enough. By using a kind of spherical integral shown in Appendix B, we have:

(89) $$\int_{R^{m}}\text{erfc}(\left\|\eta \right\|)d\eta =mB_{m}\int_{0}^{\infty }\rho^{m-1}\text{erfc}\rho d\rho$$

(90) $$=B_{m}\frac{1}{2\pi }\int_{0}^{\infty }\rho^{m}e^{-\rho^{2}/2}d\rho$$

(91) $$=B_{m}\pi^{-1/2}2^{\frac{m}{2}-1}\Gamma (\frac{m+1}{2}).$$

Then, based on Equations (88) and (91), we have:

(92) $$\varepsilon (\text{erf}(\alpha_{\text{ij}})|{\text{y}}_{i}=0)=1-{\tilde{C}}_{m}{\left(\frac{\bar{\sigma }}{r}\right)}^{m},$$

where *C˜**_m_* was given in Equation (13). Furthermore, since *r* is large enough, we can replace the condition, **y***_i_* = 0, by “when the target is well inside the *m*-ball.” Therefore, we have:

(93) $${\text{P}}_{\text{c}}=P\{\hat{a}(i)=a^{*}(i)\}=P\{\Delta J_{\text{ij}}\leq 0\}\approx 1-{\tilde{C}}_{m}{\left(\frac{\bar{\sigma }}{r}\right)}^{m}.$$

## Appendix B

In this appendix, a kind of spherical integral, used in the derivation of Equation (30), is given by [38]. Let *m* be a positive integer and *f* be a real valued measurable function defined on [0, ∞), such that *f*(*a*) ≥ 0 for all *a*. Then, we have:

(94) $$\int_{\left\|\text{x}\right\|<r}f(\left\|\text{x}\right\|)d\text{x}=\text{m}B_{m}\int_{0}^{r}\rho^{m-1}f(\rho )d\rho ,$$

for 0 ≤ *r* ≤ +∞, where ‖**x**‖ is the Euclidean norm on *^m^*, and *B_m_* is the volume of the unit ball in *^m^*.
